# Tophaceous gouty arthritis with spondylolysis: a case report

**DOI:** 10.1093/jscr/rjad689

**Published:** 2023-12-28

**Authors:** Yongsheng Ye, Fangyue Deng, Jiahui Luo, Xiongfei Huang, Xiliang Qu, Shabin Zhuang

**Affiliations:** Department of Orthopedics, Dongguan Hospital of Traditional Chinese Medicine, Dongcheng District, Dongguan City, Guangdong Province, 523000, China; Department of Orthopedics, Dongguan Hospital of Traditional Chinese Medicine, Dongcheng District, Dongguan City, Guangdong Province, 523000, China; Department of Orthopedics, Dongguan Hospital of Traditional Chinese Medicine, Dongcheng District, Dongguan City, Guangdong Province, 523000, China; Department of Orthopedics, Dongguan Hospital of Traditional Chinese Medicine, Dongcheng District, Dongguan City, Guangdong Province, 523000, China; Department of Orthopedics, Dongguan Hospital of Traditional Chinese Medicine, Dongcheng District, Dongguan City, Guangdong Province, 523000, China; Department of Orthopedics, Dongguan Hospital of Traditional Chinese Medicine, Dongcheng District, Dongguan City, Guangdong Province, 523000, China

**Keywords:** spinal gout, hyperuricemia, dual-energy CT, diagnosis, spondylolysis, treatment

## Abstract

Spinal gout is a rare occurrence, and the combination of gout with lumbar spondylolysis has not been reported. We present a unique case involving a 29-year-old male who complained of low back pain for 1 month. Computed tomography and magnetic resonance imaging revealed articular subchondral erosions and a mass in the left L5-S1 facet joints. Initially treated for a spinal infection, the patient subsequently underwent lumbar spinal canal decompression and fusion, achieving complete relief. Postoperative pathology confirmed the spinal lesions to be tophaceous gout. Dual-energy CT or biopsy can assist in confirming the diagnosis. This report discusses another rare case of tophaceous gouty arthritis with spondylolysis to be added to the literature.

## Introduction

Gout, an inflammatory arthritis resulting from monosodium urate crystal deposition, commonly affects peripheral joints, particularly the first metatarsophalangeal joint [1]. Although spinal involvement was initially perceived as rare, de Mello’s research suggests a potentially higher prevalence of tophi in the axial skeleton [2]. The literature is increasingly documenting cases of spinal gout [3]. Despite increasing recognition, the diagnosis and optimal treatment standards for spinal gout remain contentious.

We present a rare case of tophaceous gouty arthritis with spondylolysis treated at our institution.

## Case report

A 29-year-old man presented with one month of low back pain. He had no history of hyperuricemia, gout, hypertension, or kidney disease. On physical examination, he exhibited significant tenderness in the lower back area, exacerbated by movement. Neurological signs were negative, with normal lower limb strength and skin sensation. The straight leg raising test yielded negative results. Laboratory examination revealed a white blood cell count of 11.30 × 10^9^/L (reference range: 4–10 × 10^9^/L), with a neutrophilic granulocyte percentage of 70.5% (reference range: 40%–75%), and a C-reactive protein level of 81.04 mg/L (reference range: 0–10). The erythrocyte sedimentation rate was 61 mm/h (reference range: 0–15 mm/h), and serum amyloid component A was >550 mg/L (reference range: 0–10.08). The serum creatinine and serum uric acid levels were 103.5 umol/L (reference range: 57–97) and 557.0 umol/L (reference range: 202–416), respectively.

**Figure 1:**
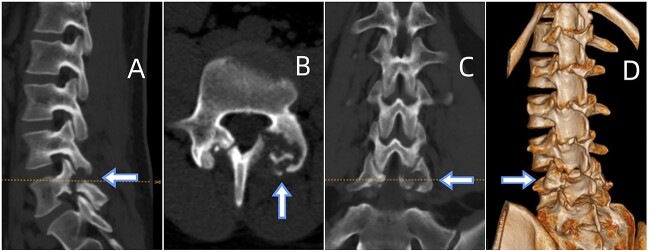
(A) Sagittal CT displaying lumbar spondylolysis at L5. (B) Axial CT revealing articular subchondral erosions and soft tissue masses at the left facet joint L5-S1. (C) Coronal CT and (D) 3D image demonstrating articular erosions and punched-out lesions of L5-S1, along with spondylolysis at L5.

**Figure 2:**
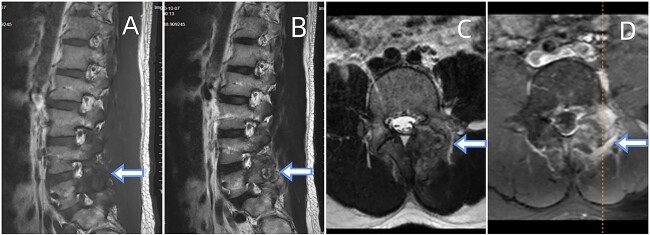
Depicts an MRI of the lumbar spine. (A) Sagittal sections of the T1-weighted. (B) A section of T2-weighted. (C) Axial section of the T2-weighted. (D) Contrast-enhanced T1-weighted. The presented images reveal an ovoid extra-dural mass measuring1.8◊1.1 cm at the subarticular area of the left L5-S1. This mass exhibits low signal intensity on T1-weighted (A) and hyperintense signal intensity on T2-weighted (B). Axial T2-weighted fat-suppressed with contrast enhancement is depicted at the L5-S1 level (C, D). The sequence illustrates a lesion that heterogeneously enhances and extends posteriorly from the left facet joint. Surrounding the L5-S1 left articular joint resection is a nodular chalky soft tissue mass.

CT showed erosions in the facet joints over left L5-S1, with soft tissue masses and sclerotic margins (Fig. 1). MRI depicted a 1.8◊1.1 cm extra-dural mass in the left L5-S1 facet joints, displaying low signal on T1-weighted and high signal on T2-weighted imaging. Gadolinium contrast highlighted heterogeneous enhancement of the lesion (Fig. 2).

**Figure 3:**
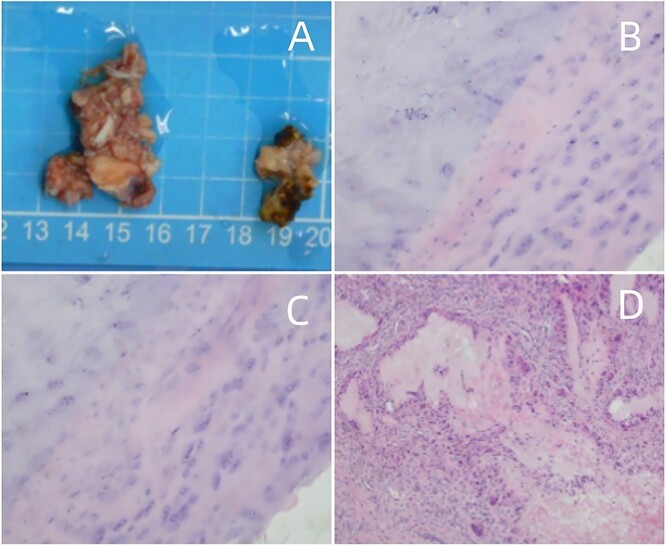
(A) Surgical pathology specimen. (B–D) Amorphous substance containing urate crystals surrounded by inflammatory cells and a multinucleated giant cell granuloma, magnified at H&E × 40.

Given ineffective antibiotics, worsening back pain, and the need for histopathological confirmation, the decision to operate was made. Debridement and transforaminal lumbar interbody fusion (TLIF) were performed for suspected infectious spondylodiscitis with an epidural abscess. Erosion of L5 lumbar spondylolysis was observed, which attributed to a white chalky mass around the left articular joint. Postoperative pathology confirmed numerous needle-shaped monosodium urate crystals, indicative of tophaceous gout. The examined mass contained fibrocartilaginous tissue, multinucleated giant cells, and amorphous crystalline material (Fig. 3), confirming tophaceous gout of the spine. After surgery, the patient received long-term urate-lowering therapy with allopurinol, febuxostat, or probenecid. Following discharge, symptoms were relieved, and repeat CT scans showed interval improvement.

## Discussion

Historically, spinal gout was considered infrequent. However, the actual incidence of spinal gout affecting facet joints and other spinal tissues may be higher than previously thought [4]. Dual-energy CT (DECT) imaging of urate deposits in the spine has revealed comparable deposits, indicating that monosodium urate (MSU) deposition in the axial skeleton could be a physiological occurrence in middle-aged men [5].

Tophaceous gout has been noted across all sections of the spine, with a predominant concentration in the cervical and lumbar regions [6]. MSU deposits can manifest in various parts of the vertebrae, frequently observed in the extradural space, followed by facet joints and vertebral bodies, but rarely in the discs [7].

Radiographic features of spinal gout are nonspecific and diverse. Intra-spinal gout tophus can be easily misinterpreted as other space-occupying lesions, such as osteomyelitis, epidural abscess, neoplasms, and synovial cysts [8]. MRI and CT stand out as the most specific imaging methods for identifying spinal gout. Lesions typically exhibit isointense or hypointense signals in T1-weighted and mostly hyperintense and isointense signals in T2-weighted MRI. Gadolinium-enhanced MRI reveals homogeneous or heterogeneous peripheral enhancement. CT shows bony erosion with sclerotic borders and normal bone density. Cardoso pioneered the use of fluorodeoxyglucose positron emission tomography (FDG PET-CT), revealing hypermetabolic activity in and around the joints in these patients [9]. A double-contour sign is observable on ultrasonography [10]. DECT in gout demonstrates excellent performance, with a pooled sensitivity of 88% (95% CI 84%–90%) and a pooled specificity of 90% (95% CI 85%–93%) [11]. However, the utility of ultrasound in detecting urate deposition in the spine is unlikely to be beneficial [12].

**Figure 4:**
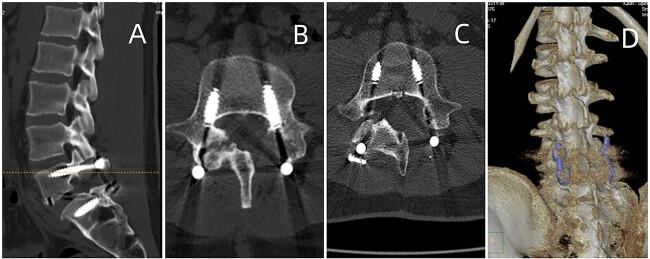
Postoperative CT of lumbar fusion surgery, depicted in (A) and (B). Two years postoperatively, DECT (displayed in (C) and (D)) reveals no urate deposition in the lumbar area.

In this study, tophi were identified as areas of low signal intensity on T1-weighted and focal high signal intensity on T2-weighted images. The tophi exhibited heterogeneous marginal enhancement with gadolinium. This case is the first to show radiological evidence of complete resolution of urate deposits in the lumbar region using DECT post TLIF and urate-lowering therapy (Fig. 4). It highlights the efficacy of DECT as a non-invasive imaging modality for assessing treatment response and elucidating the etiology of chronic tophaceous spinal gout-related back pain in patients [13].

Histopathological examination is vital for a definitive spinal gout diagnosis. Polarized light microscopy reveals crystals with pronounced negative birefringence. H&E staining in histological examination shows multiple islands of eosinophilic amorphous material surrounded by partly palisading histiocytes and a few multinucleated giant cells [14].

Degenerative spinal disease has been linked to spinal tophus development. In our case, L5-S1 spondylolysis and resulting segmental instability may contribute to spinal tophus formation. According to Wang [15], individuals with previous degenerative disc disease may have the required microenvironment for uric acid formation in the axial skeleton.

In summary, spinal gout is frequently underestimated and should be considered in the differential diagnosis of patients with back pain and an epidural mass. DECT or biopsy can assist in confirmation. Surgical decompression, whether through open surgery or percutaneous endoscopy, followed by uric acid-lowering drugs, usually leads to a favorable recovery from neurological complications.

## Conflict of interest statement

None declared.

## Funding

None declared.
